# Health promoter, advocate, legitimiser — the many roles of WHO guidelines: a qualitative study

**DOI:** 10.1186/s12961-019-0489-z

**Published:** 2019-12-05

**Authors:** Zhicheng Wang, Quinn Grundy, Lisa Parker, Lisa Bero

**Affiliations:** 1 Charles Perkins Centre, The University of Sydney School of Pharmacy, Faculty of Medicine and Health, Sydney, Australia; 2 Faculty of Nursing, University of Toronto, Toronto, Canada

**Keywords:** WHO, guidelines, global health, implementation, research utilisation

## Abstract

**Background:**

Properly implemented evidence-based clinical and public health guidelines can improve patient outcomes. WHO has been a major contributor to guideline development, publishing more than 250 guidelines on various topics since 2008. However, well-developed guidelines can only be effective if they are adequately and appropriately implemented. Herein, we aimed to explore whether and how WHO guidelines are implemented in local contexts to inform the success of future guideline implementation.

**Methods:**

Seventeen interviews were carried out between March 2018 and December 2018 with WHO guideline developers, headquarter staff, and regional and country office staff. Participants were purposely sampled from a variety of WHO guidelines and snowball sampling was used to identify regional and country office staff. The deidentified transcripts were analysed through three phases of coding, using grounded theory as the analytic approach.

**Results:**

WHO guidelines played a variety of roles in the work of WHO at all levels. WHO officers and local government officials used WHO guidelines to influence health policy. We categorised the uses of guidelines as (1) directly changing policy, (2) justifying policy change, (3) engaging stakeholders, (4) being guarantors of legitimacy, (5) being advocacy tools, and (6) intertwining with WHO’s various roles. Participants refuted the perception of the guidelines as mere lists of technical recommendations that needed to be implemented in different contexts. We found that the existence, quality and credibility, rather than the content of the guidelines, are the keys to health policy change initiatives in different local contexts.

**Conclusions:**

Used as a guarantor of legitimacy by policy-makers, WHO guidelines can be better positioned to influence health policy and practice change. Understanding the various roles of guidelines can help WHO developers package guidelines to optimise their effective implementation.

**Ethics:**

This project was conducted with ethics approval from The University of Sydney (Project number: 2017/723) and WHO (Protocol ID: 00001).

## Background

WHO has been a major developer of both clinical practice and public health guidelines [1], having published more than 250 guidelines on various topics since 2008 [1]. Evidence-based guidelines can improve clinical and public health outcomes by helping health professionals practise in the most effective manner as well as assisting policy-makers in designing optimal programmes [2].

Well-developed guidelines are thought to be effective only if they are adequately and appropriately implemented. Despite the enormous resources poured into WHO guidelines, they do not always result in effective change. One recent study found that the implementation plans included in WHO guidelines are often brief and may contain suggestions for implementation that lack evidence for their effectiveness [3]. The development of guidelines without adequate consideration of implementation may hinder the targeted audiences’ adherence to and uptake of the guidelines [4]. Without the proper implementation of guidelines by their intended users, the financial and human resources expended in the development of these guidelines by the health organisations (e.g. WHO, United States National Institute of Health, Australian National Health and Medical Research Council) will be wasted.

**Fig. 1:**
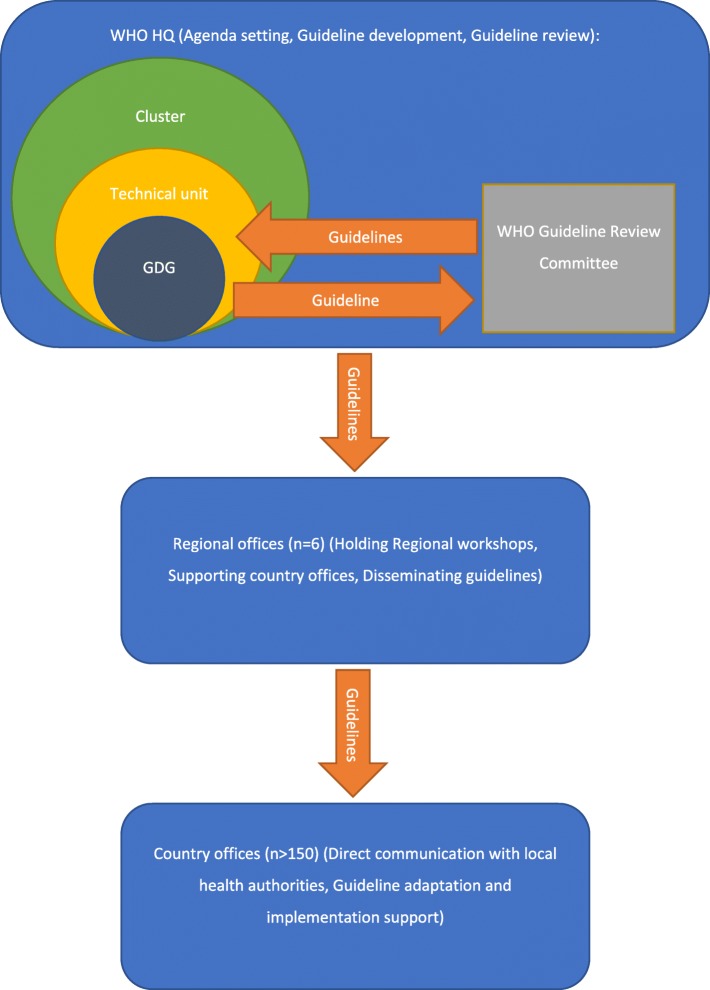
Summary of the path of WHO guidelines from development to dissemination. *GDG* Guideline Development Group, *HQ* headquarters. The WHO structure is derived from World Health Organization. The Global Guardian of Public Health [6]. The roles of each office/department of WHO involved in the guideline process are summarised in the brackets. Arrows within HQ represent the review and revision process between the GDG and the Guideline Review Committee. The Guideline Review Committee includes members who are external to HQ. GDG members are often external experts on a guideline topic assembled by a WHO technical unit for a specific guideline, not WHO staff

Since the WHO guidelines are intended for many different contexts and cultures, these factors should be considered when implementation is being planned. The local context in the case of WHO guideline implementation can be at a regional (e.g. Europe, South-East Asia or Western Pacific) or country level, or for an emergency situation (e.g. Ebola outbreak). The implementers should assess the local health culture and resources, and decide whether a particular WHO guideline is appropriate and whether adaptation, defined as “*customising (an) existing guideline(s) to suit the local context*” [5], is required. There are general structures and processes for the variety of WHO staff in disparate locations involved in developing, disseminating, adapting and implementing guidelines. The structure and functions of WHO regarding their guidelines is briefly summarised in Fig. 1 [6].

**Fig. 2:**
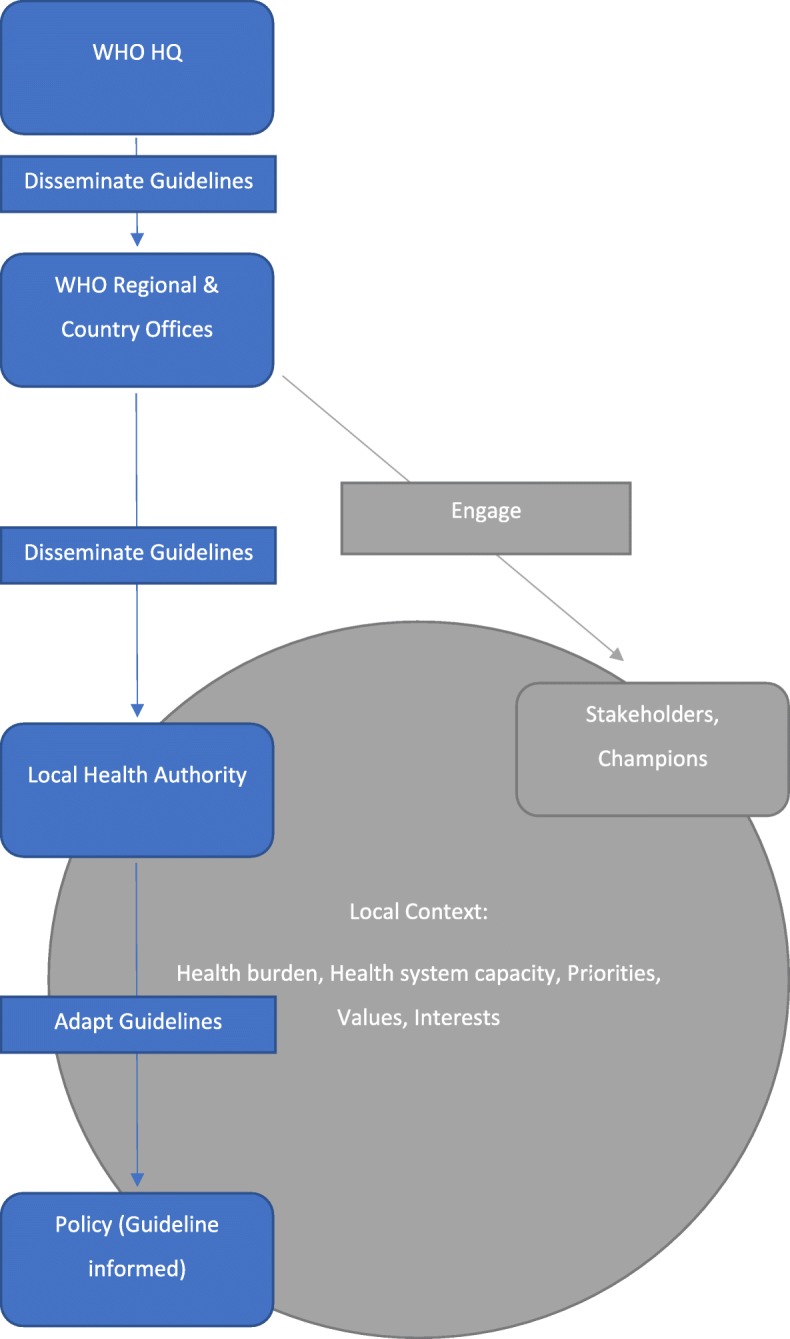
Adaptation path of WHO guidelines. A priori hypothesis of the adaptation path of WHO guidelines and processes involved. Adapted from the WHO Handbook for guideline development [7], which references the Guideline International Network (G-I-N) and the ADAPTE framework [5]

This project was guided by our understanding of the official WHO guideline dissemination and implementation process. This understanding is derived from the description in the WHO Handbook for Guideline Development [7], which references the Guideline International Network (G-I-N) and the ADAPTE framework [5]. Figure 2 summarises our a priori understanding of a typical WHO guideline implementation path.

Although WHO has reflected and improved upon its guideline development process to strengthen the quality and consistency of the content of their guidelines in the past decade [8], there has been little independent analysis of WHO’s work in improving global health through promotion, adaptation, implementation and use of WHO guidelines [9, 10]. We sought to explore whether and how WHO guidelines are implemented in local contexts to inform the success of future guidelines implementation.

### Research question

This paper is derived from a larger project exploring WHO guideline implementation. One of the key themes identified in this paper is that WHO guidelines played a variety of roles in the work of WHO at all levels. The aim of this paper is to describe the range of ways that guideline implementers used WHO guidelines in influencing local health policy.

## Methods

### Methodology, study design and rationale

We conducted a qualitative study, relying on semi-structured interviews with WHO guideline developers, staff and local implementers. The processes and practices following the development of the guidelines are often not described in the guideline documents. Tacit social processes related to whether and how adaptation to local contexts occurs are often not explicitly stated and are taken for granted. Interviews allowed ideas to emerge during the interview and for the interviewer to immediately pursue these leads [11].

This study used grounded theory methodology, which draws from the social theory of symbolic interactionism, as the theoretical approach [11, 12]. Symbolic interactionism understands that participants’ views, experiences and actions are viewed as both influenced by and influencing the process of guideline implementation [12]. A grounded theory approach aims to build an understanding (‘theory’) about the process of guideline implementation directly from the data collected [11]. A systematic and flexible approach allowed us to be open to factors that might not have been known in advance and to remain close to participants’ on-the-ground experiences.

### Ethics approval

Ethics approval for this project was obtained from the Human Ethics Committee at The University of Sydney (Project number: 2017/723) and the Research Ethics Review Committee (WHO ERC) at WHO (Protocol ID: 00001).

### Sampling and eligibility

We used purposive sampling to recruit WHO staff and guideline developers who had recent experience in assisting guideline implementation. Fifteen WHO guidelines, covering a variety of topics and WHO departments, were selected for inclusion from WHO’s database of guidelines. The named developers and WHO staff involved in the development of these guidelines were eligible for inclusion. The guideline developers that we interviewed then nominated local implementers, with whom they had worked, to be recruited into the study (snowball sampling). The structure and functions of WHO regarding their guidelines is briefly summarised in Fig. 1 [6]. We aimed to recruit participants from each of these levels of WHO offices (e.g. Headquarters (HQ), Regional and Country offices) who were involved in disseminating and/or adapting and implementing recent WHO guidelines. They were purposively sampled for maximum variation in health topics and WHO departments, according to the inclusion and exclusion criteria as discussed below.

### Inclusion and exclusion criteria

#### Guidelines

We used WHO guidelines published after 2007, following the formation of the WHO Guidelines Review Committee and the WHO Handbook for Guideline Development. Guidelines after 2007 are generally more uniform in structure [3]. The guidelines are available on WHO’s website and were downloaded according to the topics they cover. Guidelines were included if they made recommendations regarding clinical practice or public health. Guidelines were excluded if they were pictorial recommendations, charts, chapters for textbooks and/or toolkits for field use.

A total of 15 guidelines from the 10 years prior to the commencement of this project (2007–2017) were purposefully chosen to cover a diverse variety of guideline topics. This in turn covered a range of departments in WHO. WHO department structures are constantly changing and our recruitment precedes the restructuring of WHO in 2019 [13].

#### Interview participants

For each guideline, we aimed to recruit a staff member guideline developer based at HQ and a local implementer. WHO staff or guideline developers listed in the WHO guidelines and the respective local implementors they recommended were eligible to be included if they (1) had conversational level of English and (2) had experiences with the implementation of WHO guidelines (this included interactions with local implementers of the guidelines or being the implementers themselves).

### Data collection

ZW conducted the interviews between March 2018 and December 2018. Informed consent was gained from each participant prior to taking part in the study. The interviews were audio recorded and the recording was transcribed by a professional transcriptionist. Field notes were also made before, during and after the interviewing process to identify salient themes that arose and to encourage reflexivity [14].

An open-ended, semi-structured interview guide tailored to each group of participants was developed and pilot tested by the research team. It covered four discussion areas. Each area had probing questions which were asked depending on the response of the interviewee. Notes were taken during the interview in a dedicated section on the interview guide (Additional file 1). The process of guideline implementation, order of procedure (i.e. who initiates the process), barriers and facilitators, and the participants’ opinions about the process were explored in detail with the interviewees.

### Data analysis

The transcripts were deidentified using pseudonyms and removing participants’ department and location in WHO. Data analysis began simultaneously with data collection. Through this cycle, we explored ideas and issues that emerged through the analysis of early transcripts in subsequent interviews [11]. The cycle was done in the following steps:

1. Transcripts were analysed and coded using line-by-line coding in the form of open coding, describing and labelling the ideas and issues that arise in the transcripts. The initial codes and themes were used to focus subsequent interviews.
2. As more data were collected, axial coding was used as the next stage of analysis. Axial coding is the second pass of the data, and involves organising codes from different transcripts and reassembling them [15].
3. Memos were made throughout the analysis to encourage reflexivity and record the ideas that arise during the analysis process.

Data collection continued until the developed categories were saturated, that is, when new data no longer provided further insight into a particular category. Memos were a key tool in the coding process. They were written to make comparisons and to allow the researchers to piece together the process of the WHO officers’ work.

Throughout the data collection and analysis process the research group conducted fortnightly workshops to discuss interview techniques, transcripts and coding. Team members discussed salient themes that arose. This enhanced the trustworthiness of the data and encouraged the primary researcher’s (ZW) reflexivity towards the data. Feedback from these workshops challenged how the data was grouped and analysed. In turn, it resulted in the refinement of the data presentation.

## Results

We describe the multiple ways in which WHO guidelines are used by WHO staff to influence change, as synthesised from participants’ accounts in interviews. We report the process from initiation of implementation, to modifying contextual factors, to facilitating guideline implementation. We then describe the varied purposes for which guidelines are used and how participants described the roles of WHO in relation to the guidelines.

The characteristics of the interview participants are summarised in Table 1. The requests that did not culminate in interviews were all due to potential participants not responding to recruitment emails (ethics approval did not permit further emails if two were unanswered). The interviews were performed in a variety of formats (e.g. Skype (*n* = 10), WebEx (*n* = 3), WhatsApp (*n* = 2) and by telephone (*n* = 2)) depending on the availability and preferences of the interviewees. Each interview lasted 52 min on average (range: 33–61 min). ZW sent out recruitment requests to 42 individuals. ZW conducted 17 interviews with 18 participants (one interview was done with two participants simultaneously upon their request).

**Table 1:** Characteristics of interview participants

WHO Offices	No. of potential participants emailed	No. of participants interviewed
Headquarters	17	8
Regional	8	4
Country	17	6
Region of Regional and Country Office Participants	(*n* = 25)	(*n* = 10)
African Region	3	0
Region of the Americas	3	3
South-East Asia Region	5	2
European Region	7	3
Eastern Mediterranean Region	4	0
Western Pacific Region	3	2

### Guidelines used for multiple goals

Interview participants often saw WHO guidelines as auxiliary to their work as regional and country level staff. Guidelines served as a tool for staff to further WHO’s ultimate mission of policy change and improving healthcare and health systems. The regional and country level staff’s work involved, but was not dominated by, the dissemination, adaptation and implementation of guideline documents. Instead, the staff saw their work as aligned with country needs (e.g. specific requests and challenges encountered) and/or WHO’s main agenda (e.g. universal health coverage) [16]. Greg, a Regional Officer, explained the importance of the local context for directing implementation efforts:

“*Guidelines, normative guidance, and implementation, adaptation to guidelines is but one factor* [in influencing/promoting best practice]*. It’s a critical factor, but it’s but one factor. So, I think that you have to see implementation of guidances, how can we improve the health care of individuals in societies around the world? So you have to see* [health policy change] *in the context of the health insurance system, the general health system*.” (Greg, Regional Officer).

The emphasis was on the WHO’s mission for wider system level change and guidelines as simply one part of this mission. This discovery roused our interest in exploring the multiple roles of WHO guidelines in the process of improving clinical practice and health system change.

### Initiation of guideline implementation and adaptation

The publication or update of a WHO guideline was not always the main initiator of change at a local level. Hierarchical processes occurred at the regional and country offices following the publication of a guideline. These processes somewhat followed our original hypothesised steps (Fig. 2.), but the exact steps varied depending on the availability of resources to a WHO department and/or regional/country office and the local situation. Often, there was not enough “*human capacity... time and resources to plan and structure* [the implementation and adaptation process]” (Mary, HQ Staff).

Timing was very important for the initiation of the guideline adaptation process. Local events, health challenges and/or shifting political commitment were all incentives for the country to initiate contact with WHO. A country’s legislative process and health policy-making timeline were independent of WHO guidelines being published and disseminated. Therefore, incorporating the guidelines as soon as they were developed was not always appropriate in all settings. It was up to the WHO staff to build a strong case for WHO guidelines until local health authorities decided on their actions accordingly. Jessica, a Country Office staff member, described this process as “*arm*[ing] *the government with all the research of the global data and with all of the research evidences to really make the proposal a very solid one*”. Communicating the best evidence-based recommendations to local governments could rouse policy-makers’ interest until they are ready and willing to act.

The incorporation of WHO guideline recommendations into national health practice and policy was entirely up to the local health authorities (e.g. the country’s Ministry of Health). Often, WHO’s work was to communicate and promote best practice and to build a strong case for the recommendations in the guidelines. However, some countries may “*recognise the global authority or health authority of WHO and* [some] *often won’t*”, some “*largely make their own decisions*”, and others “*rely heavily on the advice from WHO and wouldn’t implement some novel technique that hadn’t been endorsed by a formal process at WHO*”, explained David, a HQ staff member. Communicating the guideline recommendations was the first step but what came next was dependent on the local context.

### Modifying contextual factors

WHO’s roles and methods were dynamic and varied depending on the specifics of health practice/policy that the countries wanted to implement or change. Often, to change national level health outcomes, system-wide/contextual changes were necessary. Guideline adherence by health professionals, and the ultimate improvement of population health outcomes, relied as much on individual knowledge and initiative as the health system contexts within which the health professionals practised. WHO officers often worked to improve these contextual factors in their efforts to improve health. A few salient examples drawn from the interviews are summarised in Table 2.

**Table 2:** Examples of WHO work to modify contextual health factors to improve guideline implementation

Contextual issue	Situation	WHO work as described by participants
Increasing medication access	A country wanted to implement a new pharmaceutical intervention according to WHO guidelines and “*These medicines are not only expensive for health systems but also for individuals*” (Greg, Regional Office staff)	Regional and country offices “*work with medicines departments, we assist the Ministry of Health, we’ve been undertaking an investment case for* [disease 1]”) to increase access and decrease costs of the new medications to both health systems and to individuals (Greg, Regional Office staff)
Raising awareness/decreasing stigma	Reducing stigma surrounding a poorly understood condition	Regional and country officers would encourage programmes “*raising awareness of all parties and healthcare providers, general population, politicians, everybody*” for the condition and treatment of the condition, and “*speaking up, talking about it — that’s the first step towards reducing stigma*” (Fiona, HQ staff)
Collectivising key populations	Reaching marginalised patient populations	Regional and Country Officers advocated for local key populations to collectivise and form networks to help each other. Sometimes, they even have key population help run clinics as “*they would rather go to these clinics, because they are more friendly*” (Helen, HQ staff)

Inadequate medication access is a well know barrier to the delivery of healthcare. It restricts clinicians’ ability to practice according to the evidence-based recommendations in WHO guidelines. In the case of new and expensive pharmaceutical interventions that are sold only by the brand name company that owns the patent, Carl, a HQ staff member explained the way he worked to decrease the cost of medications. He encouraged generic companies to apply for ‘pre-qualification’, which ensures the quality, safety and efficacy of products as they come on the market, and worked with countries to include medications into their national health insurance scheme (Carl, HQ staff). Interview participants also raised that, along with inclusion into the local public system, cost may be further reduced by “*a ceiling price being* [set for] *the originator drug, and then generic competition resulting in much reduced prices*” (Greg, Regional Office staff). These contextual modifications were all implemented to increase the availability of medications that were recommended in WHO guidelines. This work to increase medication access was both informed by the updated WHO guidelines and a prerequisite for effective guideline implementation.

Raising awareness and decreasing stigma of certain conditions was also an integral part of the work of WHO to improve contextual factors in improving health outcomes. Previous literature has described the stigma associated with people living with a variety of health conditions (e.g. HIV, hepatitis, mental health, sexually transmitted infections) [17–19]. The interview participants described that WHO works on global, regional and country levels to raise awareness about conditions and decrease stigma by holding global forums such as World Health Day, gaining “*political commitment*” at regional workshops and “*support*[ing] *Ministries of Health to think about raising awareness at a committee level*” (Fiona, HQ staff). These efforts aimed to increase understanding of the conditions regarding transmission, prognosis and treatment among governments, clinicians and the general public. Participants believed having open conversations about these conditions could increase diagnosis and treatment rates to ultimately improve patient outcomes.

Participants described collectivising key populations as another aspect of WHO’s work in improving contextual factors to improve patient outcomes. Marginalised populations were often the most vulnerable to health inequities [20]. By collectivising and forming networks of key populations to help each other, WHO worked to encourage an environment of healthcare provision that caters for these marginalised populations (e.g. the key populations helped run clinics). The increased opportunity for healthcare, in turn, can improve patient outcomes by using best practice (e.g. clinics practise according to guideline recommendations).

WHO guidelines were used to support all of these activities of WHO regional and country offices to modify contextual health factors. The improvement of these contextual factors was often beneficial to more than just the implementation of guidelines; they could also increase the capacity for health systems to achieve better health outcomes in general [21]. The roles of staff at the regional and country offices were, therefore, not necessarily focused around disseminating particular guidelines. Staff concentrated more on behaviour or policy change and improving systems toward better patient outcomes. The WHO guidelines were a tool to support this central role of regional and country offices, and were also used as a support document to justify a proposed policy change, a subsidy for medications access or a change in practice.

### Uses of guidelines

Interviewees saw benefits of WHO guidelines other than just the implementation of the recommendations. The uses of guidelines are summarised in Table 3.

**Table 3:** Uses of guidelines

Use	Quotation
To communicate evidence-based information and guidance for best practice	“*Since the WHO guidelines are not prescriptive, they are meant to really help decision-makers make informed decisions. It is not our role to make decisions for them*” (Andrew, HQ staff)
To justify and initiate policy changes	“[Guidelines are] *in two-ways as a reason to and a reason not to do stuff, but they’ll often say, we’re not going to do anything unless WHO says, we’ll wait for the WHO recommendations on this, and that’s a very common mention.*” (Mary, HQ Staff) Characterising adopting WHO guideline recommendations as following the “*gold standard*” (Olivia, Country Office staff)
To initiate advocacy programmes raising awareness regarding conditions and their treatment	A new intervention threshold needed “*advocacy programmes*” that “*sometimes take months, even a year*” (Nicole, Country Office staff)

Apart from directly informing health policy, guidelines are a way for WHO officers to communicate evidence-based information. In some situations, this information could be used to justify the initiation of policy creation/change or justify the existing health policies of a local context as WHO guidelines are seen as the gold standard. On the other hand, the change in practice may not begin until the publication of a guideline has stimulated an advocacy programme to raise awareness of a problem. Practice change may be implemented when the problem has reached a certain level of notoriety and gained the attention of the local government.

### Stakeholder engagement

Another important aspect of WHO guideline implementation was stakeholder engagement, specifically, the negotiations with stakeholders when a guideline implementation project was launched in conjunction with local health authorities. These can be seen as traditional implementation science-based methods directly linked to WHO guideline recommendations or linked to a policy change initiated by a country [22]. The contents of the guidelines or practice changes were presented to key opinion leaders in-country to seek feedback. The recommendations were often adjusted depending on the feedback to gain local support. The guidelines were only one source of information that influenced policy change, with the final accepted health practice/policy the result of input from the WHO guideline recommendations and local stakeholders/champions.

Case 1. Example of a stakeholder engagement demonstration project

**Table:** 

Jessica, a country office staff, described a case where a new medical procedure standard from WHO guidelines was introduced that went against the common established procedure in a country. WHO country officers expected local clinicians’ resistance to change; thus, “*before making the country guidelines we actually demonstrated this procedure in the* [local hospital] *and so we picked up two central hospitals and then we went there and then we set up the training and sat together with the* [specialists] *and the* [speciality] *doctors and nurses and* [allied health staff]*, so while they were all surrounding us and then … we actually used a* [patient model] *to go through and then we asked a lot of questions, if you think that it’s relevant or not? There were tonnes of questions coming from the hospital professionals and tonnes of comments — we cannot do this because of this, we cannot do that because of that. So it was really a negotiating process but over time then they started to buy it and then it was … there was a war around* [a particular procedure] *because they think, they have been practicing this for years and it has been lectured by the famous professors over the country and so for the health professionals, if we say that you should not do this, you should do it this way and they all — they will resist because it’s kind of revolting to the authorities, so it was a huge war. But, anyway, so in a nutshell that’s how we actually established good relations and also other good thinking around the feasibility over these guidelines once it’s published and then after that we also compromised for some points based on the comments from the health professionals and then finally we came up with the final guidelines.*” (Jessica, Country Office staff).	

### Roles of WHO

The role of WHO guidelines was intertwined with the work of WHO as the largest public health organisation in the world. The interviewees often alluded to different aspects of WHO’s work, which served a number of functions that worked in tandem with the guidelines (Table 4).

**Table 4:** Guidelines intertwined in WHO’s roles

Role of WHO	Relation to WHO guidelines
Communicator	WHO communicated freely available information and guidance to “*the Ministry of Health partners within the countries, but it is also available to be given to our various partners … civil society or non-state actors such as NGOs or academic universities as, for example, they are implementing a project or carrying out a research study, they can easily access and use it for their own purpose*” (Fiona, HQ staff). New and updated WHO guidelines were often disseminated in this process. Before the countries could consider implementation, the offices would aim to communicate the best available practices and recommendations
Promoter	WHO often worked to build a strong case for their guidelines’ recommendations making their recommendations specific to the country’s context. For example, this involved conducting “*not just kind of a cost benefit analysis but rather cost efficiency analysis, the value for money*” for particular programmes (e.g. medication subsidy on the national insurance scheme) (Nicole, Country Office staff). In helping conduct these economic analyses, WHO built a strong case for the country to increase medication access and, in turn, to enable practice according to guidelines (e.g. prescribing effective treatments)
Convener	WHO was a convener of different parties, holding meetings on international, regional and national levels. Often, this was done in the process of discussing and implementing a policy change related to a WHO guideline. “*Most of the meeting was sharing among the eight countries on how they, for instance, adapt the guidelines to policy — what their policies look like, sound like. How they monitor, how they communicate and advocate for the implementation. How they actually implement those guidelines. So, it was very rich sharing among country counterparts*” (Ellen, HQ staff). In convening different parties, WHO provided a forum for countries, health systems and stakeholders to learn from each other’s experience and overcome challenges together.
Authoritative source	Political role of WHO as an authoritative source of health information was demonstrated in a reciprocal way in the guideline implementation process. “*It can be the case that a WHO country office or a Ministry of Health office requests a technical assistance visit from WHO headquarters and it’s for political reasons, it’s not because someone in Geneva has more expertise than someone in the country. It’s because they feel they require the kind of force of a Headquarters person saying something which can hold sway over a Ministry of Health*” (Bill, HQ staff). This could improve the guideline’s adaptation and implementation. Conversely, the referral of guidelines as guarantors of legitimacy by countries could reinforce the authority of WHO.
Advocate	WHO’s role as an advocate to increase awareness or change public perception of particular conditions was often intertwined with guideline implementation. “O*ne of the strategies that we need to think about is, as we support Ministries of Health, is to think about raising awareness at a committee level, so that has to be part of a global advocacy, so WHO can play a role in that raising that global advocacy, political commitment*” (Fiona, HQ staff). Decreasing stigma in the public and the clinical setting was one of the ways to increase the use of guideline/evidence-based clinical care.

Note: These are roles of WHO derived from analysis of the interviews, not the strategic priorities and goals of WHO that have been described under the Thirteenth General Programme of Work (GPW13) of WHO (e.g. Achieving Universal Health Coverage, Addressing Health Emergencies, Promoting Healthier Populations) [16]

These roles of WHO are both fulfilled with guidelines as a tool (e.g. communicating evidence-based recommendations in the guidelines, engaging stakeholder in discussion on the recommendations) and performed to increase the implementation of best practice recommendations in the guidelines (e.g. cost-efficiency analysis for health insurance subsidies, HQ staff support in-country, stigma reduction programmes).

### Routes of influence for WHO guidelines

**Fig. 3:**
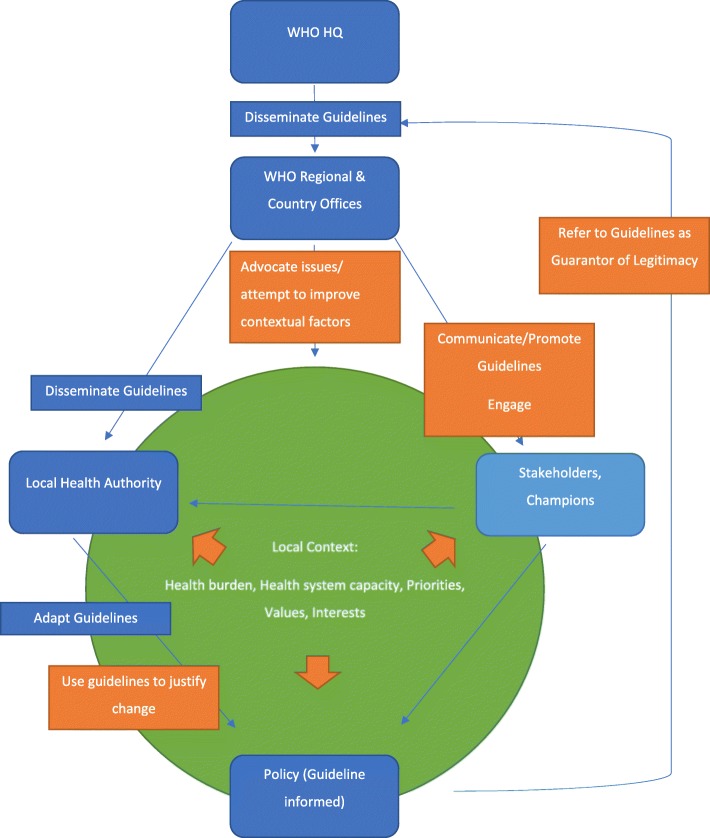
Routes of influence for WHO guidelines. Summary of the routes by which WHO guidelines can influence health policy. Original hypothesised path is in dark blue on the left. Potential ways guidelines are used (derived from the interview data) are in orange

Given the range of uses of WHO guidelines and the variety of roles of WHO that were intertwined with the guidelines, we found multiple routes by which the guidelines could influence health policy/practice in a local context. A variety of contextual factors affected WHO’s interactions with local health authorities, which in turn affected how WHO guidelines were used in different places. This was a far more complex system than the linear process we envisioned in Fig. 2. The various routes of influence that WHO and WHO guidelines could have on local health policy are described in Fig. 3. Our data show that the linear process we anticipated (shown in dark blue) is part of a more complex ecosystem that involves integrating the authority and credibility of WHO, stakeholder views, and contextual factors. The way in which guidelines are utilised varies depending on a number of factors, including but not limited to, the nature of the health issue, the local context, the level of political commitment and the acceptability of the guideline.

### Improvements to future guidelines

In discussing their experiences with WHO guidelines, the participants suggested ways to make the guidelines better fulfil their various roles. A common suggestion was to have multiple versions of guidelines or guidelines packaged in different ways to increase their reach and usability. Nicole, a Country Office staff member summarised this as:

“*I would really want to see the WHO guidelines come with either a shorter version, which is more simplified, because our guidelines, if it is like 200–300 pages, it’s kind of, especially for a national programme manager, unless somebody is really, really interested, it can be a turnoff. So, increasingly, of course, there are policy briefs that are being developed but I think so, in terms of packaging – and that* [clear] *information is extremely important.*”

Having various versions for different audiences accounting for differing levels of expertise, health literacy and time availability would potentially increase the reach of future guidelines. In doing so, WHO staff could also use the guidelines more effectively to fulfil their roles as well as those of the guidelines.

## Discussion

In the process of interviewing WHO staff about guideline adaptation, we discovered that the guidelines were used in a variety of ways to influence policy beyond their intended use to change specific practices. Unlike the a priori proposed WHO pathway for adaptation of the guidelines into local policy (Fig. 2), the role of WHO and the role of WHO guidelines was not, or at least was not limited to, the implementation of the guideline recommendations. The uses of WHO guidelines included influencing the change of contextual factors that may aid in practice improvement, using guidelines to engage stakeholders to support practice change, justifying and/or initiating policy change, communicating evidence-based information, and advocating for particular health issues.

### Models of guideline utilisation

WHO guidelines are described as evidence-based normative guidance [16]. Therefore, in extension, their use is a form of research evidence utilisation. After exploring the various ways WHO guidelines are used, we compared and contrasted with existing theory drawing from Weiss’s models of research utilisation [23] (Table 5). Weiss’ models of research utilisation were originally created to describe the use of social science research in policy-making. Thus, our findings related to health guideline utilisation offer an extension of this existing theory in a novel policy context.

**Table 5:** Comparison between models of research vs. guideline utilisation

Research utilisation	Guideline utilisation equivalent
Knowledge-driven model	Linear interpretation of WHO guideline process
Enlightenment model	Tools for advocacy and improving contextual factors
Problem-solving model	Justifying policy change
Political model	Guarantors of legitimacy
Interactive model	One source of information in stakeholder engagement
Research as part of the intellectual enterprise of the society	Intertwined with multiple roles of WHO

### Basic linear understanding of the WHO guideline implementation process

Our original linear understanding of the WHO guideline process summarised in Fig. 2 corresponds to Weiss’s knowledge-driven model of evidence utilisation [23]. Weiss describes this as “*basic research -> applied research -> development -> application*” [23]. This is an understanding of the most direct way in which empirical clinical evidence influences guideline development and guideline implementation in turn leads to evidence-based practice. The knowledge-driven model expects that, if a guideline exists, it will lead to change in practice and policy. As shown from the result of the interviews, this is one, but not the only way, in which guidelines influence policy.

### Tools for advocacy and improving contextual factors

WHO advocacy programmes often accompany or spearhead guideline implementation. These can include programmes to decrease stigma, educate patients, raise awareness of a condition and collectivise marginalised key populations to improve patient treatment. Similarly, WHO may seek to improve contextual factors such as medication availability to improve the healthcare system. These all work to indirectly improve patient outcomes and increase guideline implementation. These actions may not be directly derived from a particular WHO guideline recommendation but can be informed by the spirit of the guidelines. They are key to the success of guideline implementation. This use of guidelines has similarities to the Enlightenment Model of evidence utilisation described by Weiss [23]. Weiss envisioned the Enlightenment Model as research influencing policy when it is “*not the finding of a single study nor even a body of related studies …* [that] *affect policy. Rather it is the concepts and theoretical perspective that social science research has engendered that permeate the policy-making process*” [23].

### Justifying policy change

Weiss’s problem-solving model of evidence utilisation envisions using existing research or commissioning research to be done to solve policy problems [23]. Local health policy change may use WHO guidelines to choose or justify the adoption of evidence-based recommendations. Country-led policy change processes can use WHO guidelines or commission the adaptation of WHO guidelines into local guidelines. By referring to what is perceived as ‘gold standard’ recommendations and evidence, local health authorities use WHO guidelines to justify changing their health policies and considers these changes to be best practice. WHO guidelines are used as a point-of-reference by the local health authorities in their initiatives in improving their health policies and practices.

### Guarantors of legitimacy

WHO guidelines are often used for wider purposes than justifying or informing a particular policy change. They often serve as a guarantor of legitimacy as much as a list of technical recommendations. They can be used as tools for WHO staff to further their joint initiatives with local health authorities.

Similar to the way the WHO Essential Medicines List is viewed and used across the world [24], the recommendations in WHO guidelines are often seen by interview participants as the evidence-based gold standard. Suggestions for policy modification by local country health authorities can be legitimised if they are aligned with WHO guideline recommendations.

This role of WHO guidelines extends beyond the superficial level of WHO providing evidence-based clinical and public health recommendations. When guidelines are used as a guarantor of legitimacy, it solidifies WHO’s global leadership role in setting standards. It can be seen as self-reinforcement of the WHO’s credibility and an extension of the political role of WHO. A recent evaluation of the normative functions of WHO [25] has found that there is limited data and information available on the level and effectiveness of guideline dissemination and use. However, our findings suggest that having every WHO guideline universally followed to the word may not necessarily be the ultimate benefit of WHO guidelines. Their existence, quality and reputability, rather than merely content, are the keys to health policy change initiatives in different local contexts.

In certain contexts, countries may choose a health goal or target that is related to local health challenges and/or a current political commitment to a health issue. In these cases, they may seek to actively work with WHO and use WHO guidelines as guarantors of legitimacy to legitimise health system regulations or medication subsidies. The policy of eradicating hepatitis C is a good example of this phenomenon. In 2015, with the development of new pharmaceuticals (i.e. direct-acting antivirals) that come closer to curing the condition than existing medicines, many countries joined WHO’s commitment for the elimination of hepatitis B and C as a public health threat by 2030 [26]. This is a huge commitment, especially for countries with a high disease burden, given the high cost of the new treatments. In the context of this agenda, countries have sought to push regulations and medication subsidies. To legitimise these subsidies, WHO guidelines are often referred to as guarantors of legitimacy. This is similar to Weiss’s political model of evidence utilisation as the guideline has been used to support a political position that has already formed [23].

### Stakeholder engagement

In guideline implementation, stakeholder engagement is essential to the success of the implementation project. Recommendations may be modified depending on the local values and preferences. Local health policy-making is dependent on a variety of sources of input, of which WHO guidelines is only one. This can even be expanded to regional workshops where different countries may be brought together to share their experiences and offer each other solutions [27]. Holding regional/national workshops and engaging stakeholders in the implementation process corresponds with Weiss’s interactive model of evidence utilisation [23]. This can be formal discussions or even a demonstration project (see Case 1). Using guidelines as the initiator of discussion welcomes feedback form local stakeholders. By getting their feedback, the final adapted guideline would be more likely to have stakeholder support during implementation as well.

### Intertwined with multiple roles of WHO

The last model of evidence utilisation described by Weiss is Research as Part of the Intellectual Enterprise of the Society [23]. This is particularly interesting as Weiss envisioned research as not only influencing policy, but the converse also being true — current society, events and problems can influence how and what research is done, and what evidence is produced. It is all a part of “*the interconnected intellectual enterprise*” [23]. In the realm of WHO guidelines, this is demonstrated in their responsiveness to the needs of countries and current events. Regional and country offices can request for a guideline to be developed if they deem it necessary. However, more often, the guidelines themselves are also intertwined with multiple roles of WHO depending on the needs of the local context.

Guidelines are used in a variety of models as described above. Whether and how a country follows WHO guidelines or initiates any health policy change is dependent on the local situation and political will at a particular time. In adapting to the local situation, WHO regional and country offices intertwine WHO guideline recommendations into different parts of their programme of work. The guidelines help WHO fulfil their various roles, but those roles are also shifting in tandem with the guideline needs.

### Implications

Recognising the variety of roles that WHO guidelines play in influencing health policy and practice can help initiate innovative ways to boost guideline utilisation. By catering to the needs of different contexts, guideline developers, and regional and country officers can better utilise the guidelines to their full potential. Raising awareness of these various roles can broaden the perception that guidelines are merely lists of recommendations that need to be implemented.

With such a variety of uses for guidelines in different contexts, packaging WHO guidelines in multiple and diverse ways could be beneficial for their utilisation. Currently, WHO guidelines are typically published online and in print as a book that is often more than 100 pages long. It could be impractical to use them effectively to fulfil WHO’s various roles. Guideline operationalisation tools (e.g. recommendation summaries, decision charts) and different guideline versions for different audiences (e.g. separate documents for clinicians, policy-makers and patients) could widen the scope of WHO guidelines to fulfil their diverse roles. Having various versions that are tailored for different expertise levels and for the needs of diverse audience groups would increase the accessibility of the recommendations.

WHO is in the process of improving their guideline development to incorporate more aspects in their evidence-to-decision frameworks [28]. Including characteristics such as sociocultural acceptability, feasibility and health system considerations in the guideline development would make the content of the recommendations more adaptable. However, the results of this study show that the contents of the guidelines are already often seen as the gold standard. We suggest that prioritising and optimising how these contents are packaged in derivative products is also needed to help both WHO and WHO guidelines better fulfil their roles. This would improve the reach of the guidelines while also making them more user friendly for both WHO region and country level officers as well as end-users everywhere.

The multiple roles that WHO guidelines play may also have implications for national bodies that develop guidelines. National guidelines must also be implemented in multiple settings with different local contexts. By understanding health guidelines as more than a set of technical recommendations, guideline developers and implementers can leverage their other social functions to influence public health and clinical practise. For example, a national dietary guideline could be the impetus for a national advocacy programme, justify policy change requiring public school canteens to serve a certain variety of food, or drive stakeholder engagement to adapt the guideline to different clinical and social contexts within the county. Ultimately, using guidelines in multiple ways can better justify the large investments that are needed for their development.

### Strengths and limitations

This study was the first interview study of WHO officers, to our knowledge, to explore the adaptation and implementation of WHO guidelines. The study accessed a variety of WHO guidelines in order to discover the many ways different implementers used the guidelines during adaption and implementation. Due to the limitations in time and resources as well as the difficulties of international recruitment of WHO officers, we could only interview a relatively small sample of participants. WHO regional offices for the Eastern Mediterranean and Africa were not represented in the interviewees we were able to recruit and therefore no country offices in these regions were covered in this project. The missing perspectives may be able to shed more light on the variety of methods that the officers use to diversify the use of WHO guidelines. Future studies can explore how guidelines are used in each of the roles described here and regional/departmental differences can be further differentiated.

## Conclusion

WHO guidelines play a variety of roles in the work of all levels of WHO offices. The perception of the guidelines as lists of technical recommendations that need to be adapted and implemented in every different context is limited in scope. This project has explored the intricate ways in which the guidelines are intertwined in WHO’s work at every level. Seen as a guarantor of legitimacy by many countries in the world, WHO guidelines can be better positioned to influence health policy and practice change if their various roles are better understood. Packaging future WHO guidelines with operationalising guidance and producing multiple versions for the variety of WHO’s audiences would likely help the guidelines fulfil their roles more effectively.

## Supplementary information

**Additional file 1.** Interview guide and preamble.

## Data Availability

The datasets generated and/or analysed during the current study are not publicly available due to privacy concerns.

## Abbreviation

HQ: headquarters
