# Supplementary material for: Health promoter, advocate, legitimiser — the many roles of WHO guidelines: a qualitative study
Source: Health Res Policy Syst. 2019 Dec 5;17:96. doi: 10.1186/s12961-019-0489-z (PMC6896683; doi:10.1186/s12961-019-0489-z)
Supplement: Supplementary file 1 — Additional file 1. Interview guide and preamble. [file 12961_2019_489_MOESM1_ESM.pdf]

Zhicheng (Jeff) Wang, Charles Perkins Centre, The University of Sydney

World Health Organization Guideline Adaptation: An interview study – Individual interview guide

For WHO headquarters' staff and members of the guideline group (e.g. chair)

Preamble: Good morning (afternoon). My name is Jeff. I am a PhD student from the University of Sydney, Australia (working with Prof. Lisa Bero and Dr Susan Norris (WHO GRC secretariat)). Thank you for taking your time for this interview. This interview is about your experience with the adaptation and implementation of WHO guidelines. I would like you to feel comfortable with saying what you really think and how you really feel. As I advised you in the consent form I will be taking notes, and I would like to record this interview. The purpose of this is so that I can get all the details but at the same time be able to carry on an attentive conversation with you. I assure you that we will not publish your name, job title, or local organization name. However, we may directly quote what you shared in the interview and the country where you work.<br>I assured you that all measures will be taken to ensure your confidentiality, but that as a qualitative study where direct quotations may be used in disseminating the research, complete anonymity cannot be guaranteed. All quotations will be made anonymous (under pseudonyms) or will be "characterized" in language unidentifiable to the reader.<br>Your participation in the study is voluntary and you are able to withdraw from the study at any time without consequence<br>Do you have any questions about what I've said? Do you consent to be recorded? I will now turn on the tape.		
Discussion area	Probing questions	Notes
1) Describe your experience implementing the last WHO guideline you worked on.	• Was implementation considered during the guideline development process?<br>• Tell me about the process of guideline dissemination after their development.<br>• How was the implementation process initiated?<br>• Who in the WHO headquarters and local offices are responsible in managing the implementation process?<br>• What is the role of local implementers in the process?	

2) What worked?	• What are some facilitators to the process?	
3) What didn't?	• What are some barriers to the process?	
4) What insights did you take away from the process? What might you do differently?	• How do you think the implementation and adaptation process can be improved?<br>• What can we add to the guideline development handbook to improve the implementability and adaptability of future guidelines?	

Zhicheng (Jeff) Wang, Charles Perkins Centre, The University of Sydney

For local implementers

Preamble: Same as above		
Discussion area	Probing questions	Notes
1) Describe your experience the last time you implemented a WHO guideline.	• How was the implementation process initiated?<br>• Who in the WHO headquarters and local offices did you work with in managing the implementation process? What were their roles?	
2) What worked?	• What are some facilitators to the process?	
3) What didn't?	• What are some barriers to the process?	
4) What insights did you take away from the process? What might you do differently?	• How do you think the implementation and adaptation process can be improved?<br>• What can we add to the guideline development handbook to improve the implementability and adaptability of future guidelines?	
